# Ommaya Reservoir Insertion: A Technical Note

**DOI:** 10.7759/cureus.7731

**Published:** 2020-04-18

**Authors:** Stephen T Magill, Winward Choy, Minh P Nguyen, Michael W. McDermott

**Affiliations:** 1 Neurological Surgery, University of California San Francisco, San Francisco, USA

**Keywords:** ommaya, reservoir, image guidance, intrathecal, chemotherapy, intraventricular

## Abstract

Ommaya reservoir insertion is an elective neurosurgical procedure to deliver repeated intraventricular therapy, but placement can be complicated by malposition of the catheter, clogging, infection or poor postoperative cosmesis. Here, we describe the technique used by the senior author for accurate placement including preassembly of the reservoir and catheter, and recessing of the reservoir so that others may consider the technique for their practice. Results in a consecutive series of 27 Ommaya placements were reviewed. Catheter tip placement accuracy, complications and surgical times were reported. Indications were leptomeningeal cancer or infection. Postoperative imaging showed the catheter tip was located in the frontal horn (96%) or body (4%) of the ipsilateral lateral ventricle. The median surgical time was 36 minutes (range 17-63 minutes). There were no parenchymal or subarachnoid hemorrhages. Infections occurred in 7% (n=2) of cases, and both infections presented greater than 60 days postoperative. In conclusion, we have found that image guidance can optimize accuracy in placement, that preassembly of the reservoir and catheter may be used with a 25-gauge spinal needle stylet to minimize risk of clogging during placement, and that recessing of the reservoir produces the best aesthetic result.

## Introduction

Ommaya reservoir is a valuable neurosurgical tool to deliver regular intraventricular therapy and sample the cerebrospinal fluid (CSF) without the need for serial lumbar punctures [1-4]. Since its first description in 1963, multiple papers have been published about techniques for the insertion of Ommaya reservoirs using free-hand, frame-based and image-guided methods, all of which have demonstrated success [2-4]. Improper catheter positioning and poor reservoir placement can lead to neurological complications, nonfunctioning reservoirs and the need for reoperation to reposition [2,5]. Postoperative infection, typically with gram-positive skin organisms, occurs in 5%-8% of patients and stratifies into infections occurring around the time of placement, and delayed infections, typically after recent access of the reservoir [5,6]. With the increasing prevalence of precision-based medicine, including immunotherapy and small molecule inhibitors, cancer patients are living longer and more patients are surviving with late-stage leptomeningeal dissemination, increasing the need for effective drug delivery to the CSF [7-10]. Here, we document our technique for image-guided insertion of Ommaya reservoirs and review our results using this technique.

## Technical report

Methods

Study Design, Setting, Size and Participants

A series of consecutive surgical procedures for the placement of an Ommaya reservoir with image guidance from 2015 to 2020 by the senior author were reviewed. The step-by-step technique was recorded with photographs for illustration. Results of catheter tip position and surgical, positioning and anesthetic times were recorded in minutes. Early and late infections were documented and recorded. This study was approved by our institutional review board (IRB #15-17500). The patient whose images were included provided consent for publication. The institutional review board did not deem consent necessary for the chart review portion of the study.

Surgical Procedure: Planning

**Figure 1:**
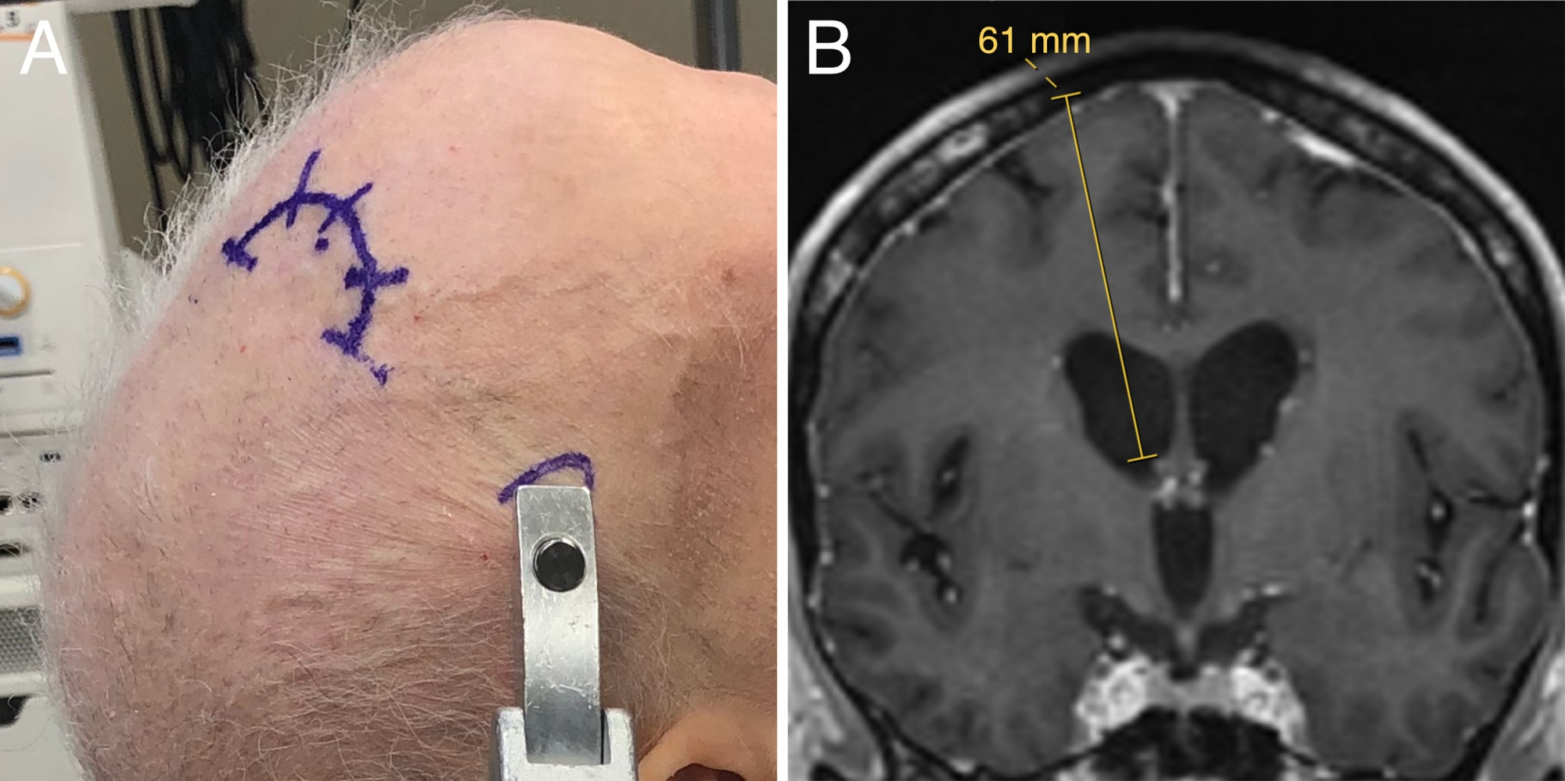
Positioning. (A) The patient is positioned supine in a Mayfield head holder. The incision is based posteriorly, which cuts the cutaneous scalp sensory nerves to the skin over the reservoir, which numbs the area needed for accessing the reservoir, minimizing pain for the patient when it is accessed during the first couple months after placement. (B) A trajectory is planned from the middle of the diploic space to just dorsal to the foramen of Monro.

Preoperative MRI scans of brain with and without contrast were performed to document size of the ventricular system, exclude parenchymal lesions along the path of the proposed trajectory and provide a volumetric study for use with the image-guided neuronavigation system. Fiducials are not required for patients with smooth skin that lacked wrinkles. Prior to surgery, we plan an ideal trajectory using the neuronavigation software. Using coronal images, we measure the distance from the middle of the diploic space to the base of the frontal horn near the foramen of Monro to measure the expected catheter length (Figure 1). The middle of the diploic space is selected to account for the thickness of the Ommaya reservoir and the effect of recessing the reservoir (described below).

Surgical Procedure: Positioning

Under general anesthesia, the patient is positioned supine on the operating table and the head placed in a Mayfield head holder (Integra LifeSciences, Princeton, NJ) with the neck slightly flexed on the chest and the head neutral in relation to the neck (Figure 1A). The neuronavigation reference arc is placed to the patient’s left with the top of the array in line with the mid-point of the Mayfield pin headrest to keep it away from the surgeon’s working area. The accuracy of the registration of imaging to physical space is confirmed with anatomic landmarks and/or fiducials. A paramedian trajectory to the frontal horn of the lateral ventricle is selected with a frontal entry point anterior to the coronal suture that terminates in the frontal horn of the lateral ventricle just dorsal to the foramen of Monro to keep the tip of the catheter away from the choroid plexus (Figure 1B). The entry point is marked on the skin and a posteriorly based semi-circular incision is marked (Figure 1A). Importantly, basing the incision posteriorly results in transection of superficial scalp sensory nerves coming from the supraorbital foramen. This causes the skin over the Ommaya reservoir to be numb for the first few months postoperatively, which decreases the patient’s pain when the reservoir is accessed.

Surgical Procedure: Equipment Preparation

**Figure 2:**
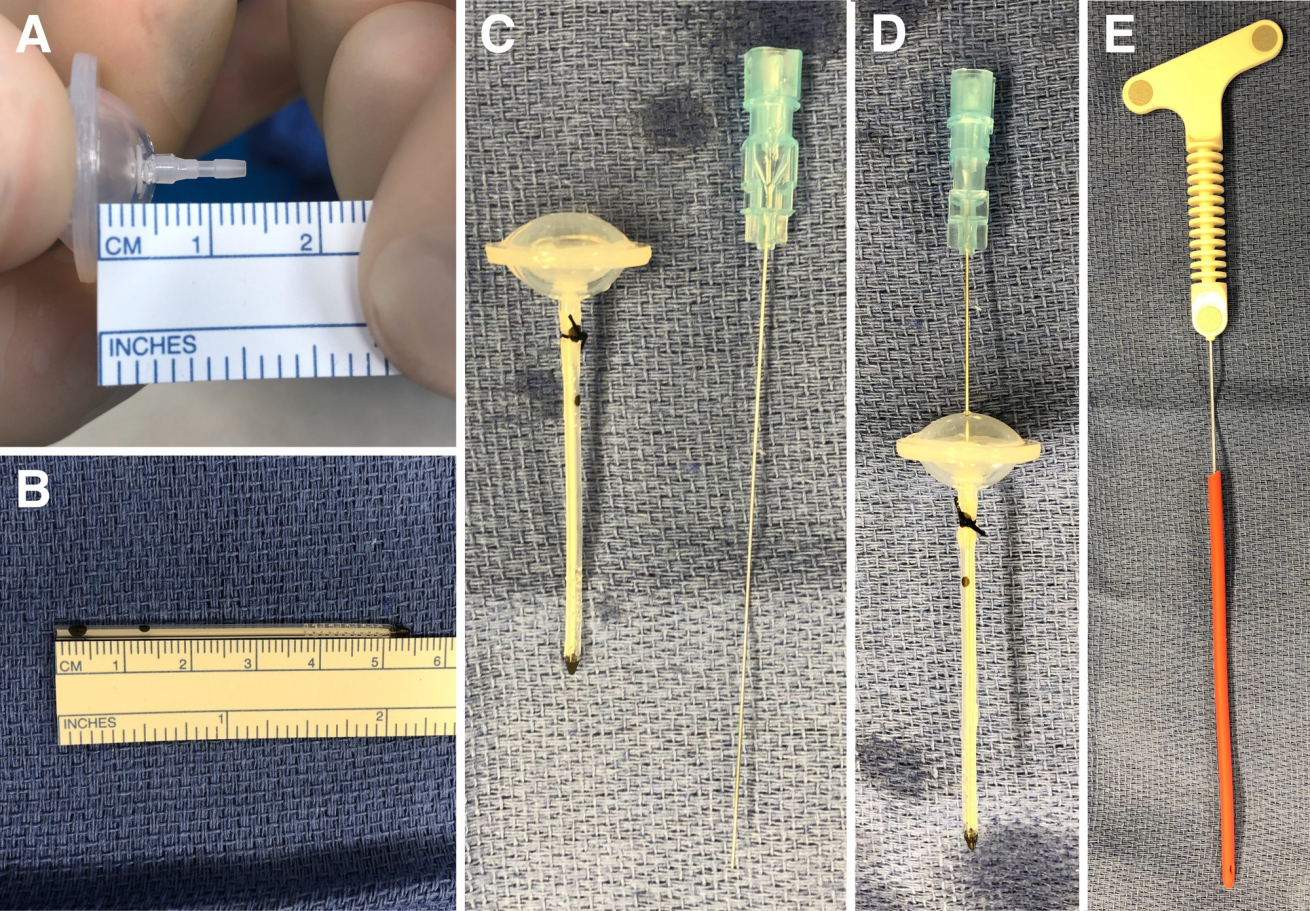
Equipment setup. (A) The Ommaya reservoir has 7 mm between the flange, which is placed in the diploic space, and the proximal connection point for the catheter. (B) Given that the planned length (Figure 1B) was 61 mm, the catheter is cut to 54 mm, which achieves the desired distance from the Ommaya flange to the catheter tip. (C) The Ommaya reservoir is preassembled with the catheter and secured with a silk suture. (D) A 25-gauge spinal needle is placed into the reservoir to the tip of the catheter and used as a stylet for placement. (E) A #9 French red rubber catheter is placed over an image-guided stylet, which will be used to create the transcortical path for reservoir placement.

The surgical field is prepared and draped in a sterile fashion. On the scrub table, the ventricular catheter is cut to the planed length and assembled for insertion (Figure 2). The catheter is cut 7 mm short of the planned trajectory to account for the distance from the flange of the Ommaya reservoir to the proximal limit on the tip that connects to the catheter (Figures 2A, 2B). This allows the individualized ideal length to be achieved once the reservoir is recessed into the diploic space. The reservoir and catheter are connected and secured with a nonabsorbable braided nylon suture (Figure 2C). A 25-gauge spinal needle is then inserted through the center of the dome to the end of the catheter to act as a stylet for insertion through the brain (Figure 2D). Finally, a #9 French red rubber catheter is cut and placed over an image-guided stylet to be used for creating a transcortical path (Figure 2E).

Surgical Procedure: Placement

**Figure 3:**
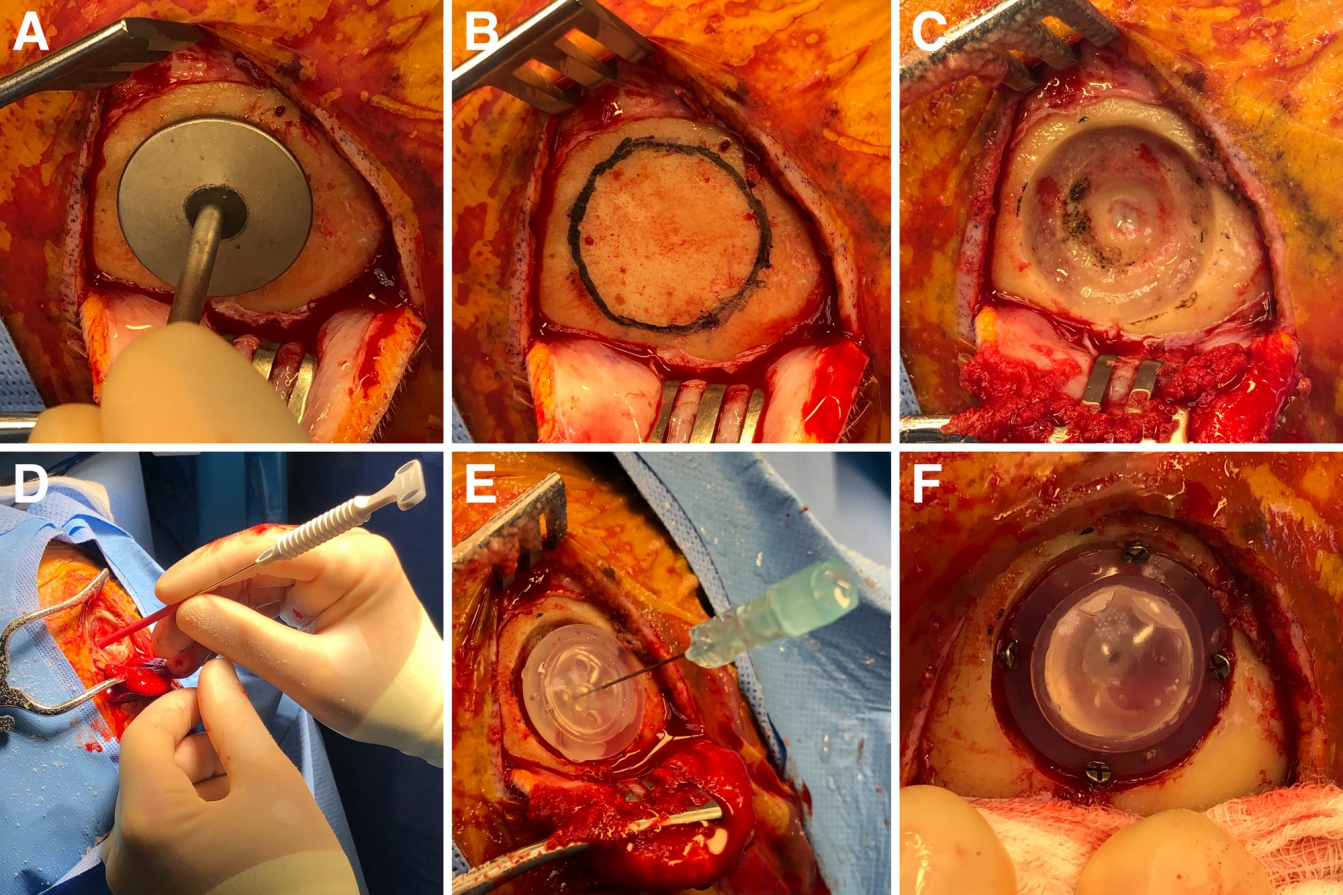
Intraoperative procedure. After raising the scalp and periosteal flap, (A) the template is used to make out the size of the burr hole. (B) Planned burr hole. (C) The outer rim of the burr hole is drilled partial thickness into the diploic bone, while the center of the burr hole is taken to dura and 3-4 mm of dura is exposed. (D) Once the dura and pia are coagulated and opened, the red rubber catheter is used to create a transcortical path with image guidance into the frontal horn of the lateral ventricle to achieve accurate placement. Cerebrospinal fluid flow after removal of the stylet confirms the correct position. (E) The preassembled Ommaya with the spinal needle acting as a stylet can be gently passed down the cortical trajectory created by the red rubber catheter. (F) The reservoir is secured in place with 5 mm screws angled into the adjacent skull.

The skin and pericranium are reflected posteriorly in a single layer, and the frontal bone anterior to the coronal suture is exposed. A template with a diameter equal to the base of the burr hole designed for an Ommaya reservoir is used to mark out the planned burr hole to allow for a recessed positioning of the reservoir (Figures 3A, 3B). A round cutting burr is used to create a two-layer burr hole that mirrors the topology of the Ommaya reservoir. This is easily done by drilling down to the dura in the center of the burr hole, and then creating levels within the diploic space to accommodate the reservoir (Figure 3C). Once the dura is exposed, it is opened in a cruciate manner and a 3-4 mm incision is made in the pial surface (Figure 3C).

**Figure 4:**
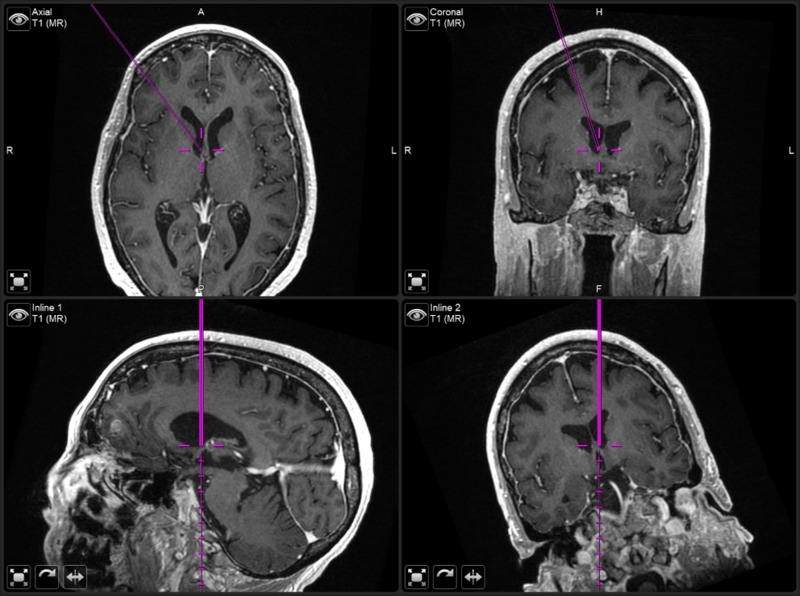
Neuronavigation trajectory. Neuronavigation screenshot showing axial (top left), coronal (top right), and inline/trajectory views (bottom row) of the image-guided stylet at the ideal position slightly dorsal to the Foramen of Monro.

Next, the image-guided stylet is used to pass the #9 French red rubber catheter under image guidance into the ventricle (Figures 3D, 4). The stylet is withdrawn to confirm the presence of flow and the catheter removed. Creating this initial path with the red rubber catheter, rather than the Ommaya catheter, decreases the chances of parenchyma entering into the Ommaya catheter and clogging it. The Ommaya reservoir is introduced softly along the same path holding the base of the spinal needle (Figure 3E). The needle is removed. While holding the rim of the Ommaya reservoir against the bone, the dome is depressed to confirm clear CSF return in the Ommaya. The Ommaya is then secured using four diagonally placed 5 mm screws (Figure 3F). The wound is irrigated with 3% betadine solution and 250 mg of vancomycin powder is placed in the subgaleal space. The galea is closed with 3-0 Vicryl® suture (Ethicon Inc, Somerville, NJ) on an RB-1 needle and the skin with 4-0 Novafil™ suture (Medtronic, Minneapolis, MN) on a P-12 needle. Bacitracin ointment is applied followed by a dressing. Accessing of the Ommaya reservoir for intrathecal therapy is allowed beginning on postoperative day 1.

Results

The results of Ommaya placement using the described method were reviewed by the senior author. In a five-year period, 27 reservoirs were inserted (Table 1). The median age was 54 years and 70% of the patients were female. Indications for insertion were leptomeningeal cancer or infection requiring chronic intrathecal antifungal therapy. Primary cancers were: lymphoma (n=12, 44%), breast cancer (n=8, 30%), melanoma (n=4, 15%) and lung cancer (n=2, 7%). One patient (4%) had an intracranial mycosis fungoidies infection that required long-term intrathecal antifungal therapy, and the infectious disease team requested an Ommaya for intrathecal therapy. The median anesthetic time was 19 minutes (range, 9-53 minutes). Median setup and positioning time, as defined by the time between when anesthesia recorded that they were ready and the time of the skin incision, was 20 minutes (range, 0-39 minutes). Finally, median surgical time, defined as the time from the incision to having the patient out of the head holder and repositioned for extubation, was 36 minutes (range, 17-63 minutes).

**Table 1:** Patient characteristics and operative results. SD, standard deviation.

Patients (no.)	27
Median age in years (range)	54 (31-77)
Number female	19 (70%)
Diagnosis	
Lymphoma	12 (44%)
Breast cancer	8 (30%)
Melanoma	4 (15%)
Lung cancer	2 (7%)
Infection	1 (4%)
Operative times	
Anesthesia time in minutes	
Mean, SD	20 (9.0)
Median, range	19 (9-53)
Setup time in minutes	
Mean, SD	20 (8.6)
Median, range	20 (0-39)
Surgical time in minutes	
Mean, SD	35 (9.6)
Median, range	36 (17-63)
Operative results	
Catheter placement (n=24)	
Ipsilateral ventricle, frontal horn	23 (96%)
Ipsilateral ventricle, body	1 (4%)
Infection	
Less than 60 days postoperative	0 (0%)
Greater than 60 days postoperative	2 (7%)

**Figure 5:**
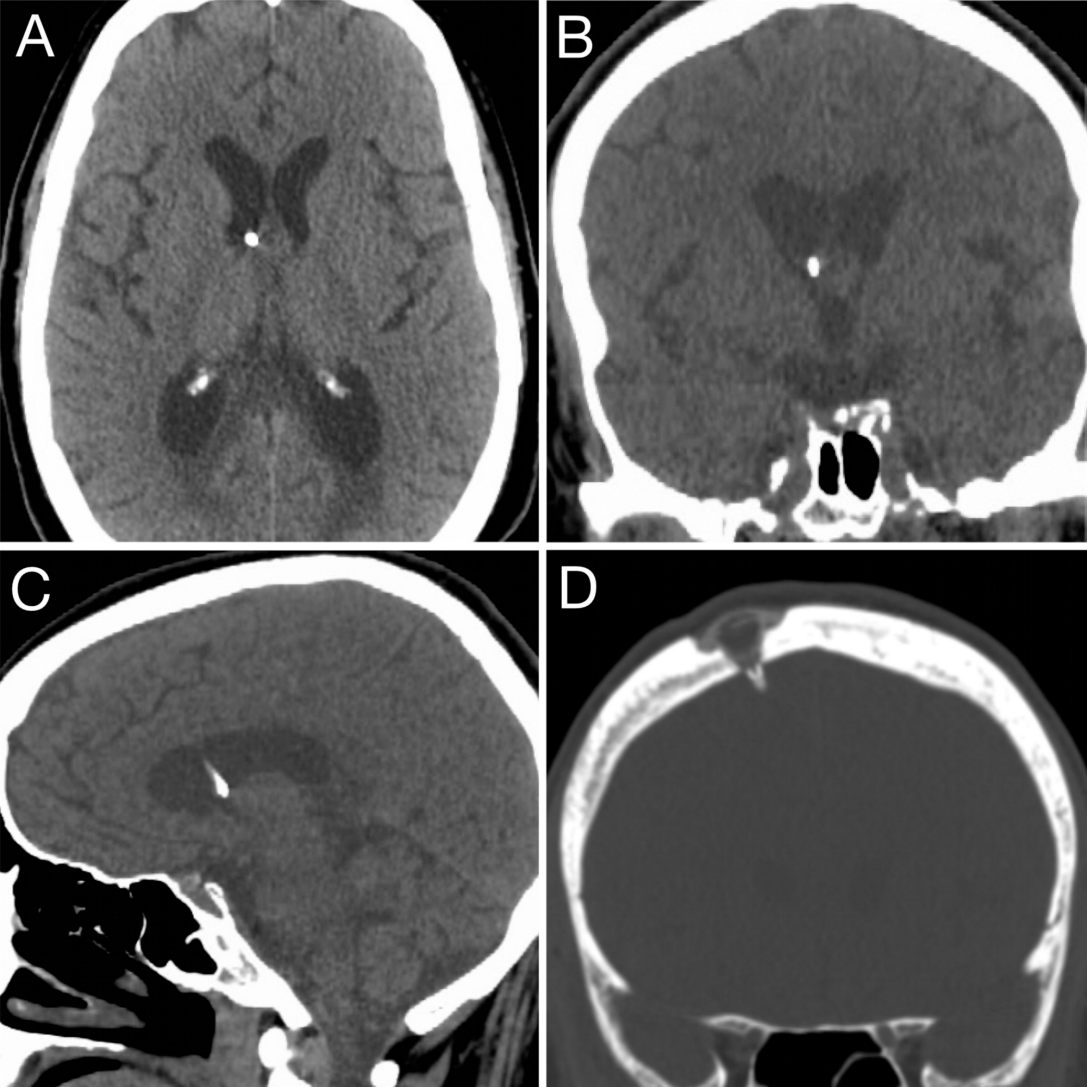
Postoperative imaging. Postoperative noncontrast CT scan showing ideal catheter placement in (A) axial, (B) coronal and (C) sagittal planes. (D) Bone window coronal CT scan showing the recessed Ommaya reservoir and smooth surface of the skin over the reservoir.

For the 24 patients with postoperative imaging (Figure 5), all catheter tips were in the ipsilateral lateral ventricle, with the majority in the frontal horn (n=23, 96%) and one catheter (4%) in the body. There were no parenchymal or subarachnoid hemorrhages. Infections occurred in 7% of cases. No infections occurred within 60 days of reservoir placement. The two (7%) infections occurred greater than 60 days after placement, grew gram-positive bacteria and required removal of the Ommaya reservoir. Recessing of the Ommaya reservoir within the diploic space resulted in excellent cosmesis, with a smooth skin surface (Figure 5D) and no visible protrusion of the reservoir.

## Discussion

This technical note describes the technique developed by the senior author for elective insertion of Ommaya reservoirs. Postoperative complications after Ommaya placement, such as clogging, poor placement or infection, may require revision surgery, which can delay life-prolonging chemotherapy while the patient goes through the additional surgery and recovery.

There are several key nuances that we believe are particularly useful in achieving successful reservoir placement. First, we strongly recommend using image guidance with a neuronavigation system for placement. Image guidance aids in ventricular access and ensures accurate placement [4]. Thus, we feel that the small increase in setup time is worth the increased accuracy and safety, which is reflected by our success rate with no patients needing revision surgery for misplacement and all catheters being located in the ipsilateral lateral ventricle. Given the accuracy of our technique, we no longer recommend routine postoperative imaging. Second, preassembling the reservoir and catheter on the scrub table avoids the challenge of securing the catheter to the reservoir while the catheter is in the brain, which could lead to improper positioning of the catheter or damage to surrounding brain as the catheter may be incidentally pulled, moved or torqued while attempting to securely suture the catheter and reservoir together. Third, the use of a 25-gauge needle as the central stylet avoids compression of the dome when inserting the device. If the needle/stylet is not used, even mild compression on the dome may cause friction and negative pressure on adjacent white matter during placement, which risks aspiration of white matter into the device. In addition to damaging the white matter, any aspirated material may clog the catheter. Fourthly, recessing the device into the bone, rather than placing it on the skull, minimizes the cosmetic deformity that the reservoir can produce, which many patients have expressed concern about during preoperative conversations. Recessing the valve does not adversely affect reservoir access or function. During informal discussion with our oncology colleagues who administer chemotherapy through the Ommaya, they reported no difficulty in identifying or accessing the recessed reservoir.

Finally, infection of the device is a terrible complication for the patient, as it may delay therapy. We have not had any infections within 30 days of placement since we started placing vancomycin powder into the subgaleal space around the device during closure, but interpretation of this observation is cautioned, as this study size is not sufficient to study infection rates given their overall low incidence. Our delayed infection rate, which is likely due to contamination during access, is comparable to prior reports [5,6]. To minimize this risk, we provide our patients with an oral antibiotic, typically clindamycin unless the patient is allergic, and recommend they take a single oral dose one hour prior to accessing the device. Based on our clinical judgment, we also recommend an antibiotic prophylaxis when patients with implanted Ommaya reservoirs have dental work performed, although again, no adequately powered studies have investigated the necessity of prophylactic antibiotics in this population.

## Conclusions

The described technique for Ommaya reservoir insertion addresses the potential risks of misplacement, clogging, infection and poor cosmesis that the neurosurgeon may encounter during placement. We suggest that image guidance should be used to optimize accuracy in placement, preassembly of the reservoir and catheter should be used with a stylet to minimize risk of clogging and recessing of the reservoir to produce the best aesthetic result.
